# Special Issue “Prostate Cancer: Recent Advances in Diagnostics and Treatment Planning”

**DOI:** 10.3390/jcm11226823

**Published:** 2022-11-18

**Authors:** Theodoros Tokas

**Affiliations:** 1 Department of Urology and Andrology, General Hospital Hall i.T., Milser Str. 10, 6060 Hall in Tirol, Austria; ttokas@yahoo.com; Tel.: +43-(0)50504-36310; 2 Training and Research in Urological Surgery and Technology (T.R.U.S.T.)-Group, Milser Str. 10, 6060 Hall in Tirol, Austria

This editorial of the Special Issue “Prostate Cancer: Recent Advances in Diagnostics and Treatment Planning” aims to draw more attention to the broad and diverse field of prostate cancer (PCa) diagnosis and the utilization of different diagnostic means to improve clinical decision-making and treatment strategy planning. PCa is the second most frequent malignancy in men [1]. Tumor aggressiveness varies, ranging from non-aggressive tumors that may be safely monitored to poor prognosis tumors only suited for palliative treatment. Undoubtedly, new imaging modalities such as magnetic resonance imaging (MRI) and positron emission tomography (PET) with targeted tracers are more sensitive than conventional imaging [2] and may result in stage migration and a natural inclination toward altering clinical management. In contrast to other cancers, the PCa community acknowledges that precision medicine has developed more slowly [3]. Genetic counseling and germline testing can aid in the early detection and management of PCa. Biomarkers based on urine, serum, and tissue increase PCa patient detection and facilitate risk stratification.

Indications for prostate biopsy can be determined with the aid of MRI, which is also essential for local staging. When combined with clinicopathological information, MRI results in a more accurate prognosis, which may help with tailored patient care [4]. In the case of localized PCa, MRI findings are associated with clinically relevant long-term oncologic outcomes. The diagnosis of clinically significant PCa is improved by targeted biopsies, as routine transrectal ultrasonography is not always accurate. Additionally, the evidence supporting the addition of MRI-targeted biopsies to systematic biopsies necessitates a review of the active surveillance (AS) inclusion criteria and a shift in research focus away from one-size-fits-all protocols and toward more flexible and personalized risk-based AS approaches [5]. On the other hand, modern, less expensive ultrasound-based techniques can deliver high-quality imaging in the absence of an MRI [6,7,8]. 

Prostate-specific membrane antigen (PSMA) PET has been adopted for staging aggressive tumors. Compared with traditional imaging, PSMA PET offers a reasonably good sensitivity for detecting regional and extrapelvic metastases. Additionally, it can play a significant part in the early diagnosis of extraprostatic disease and help with surgical planning. Furthermore, PSMA PET has been shown to be a valuable technique for planning definitive radiation therapy in patients who have not yet received treatment [9]. Furthermore, even at low PSA levels, PSMA PET is highly effective at detecting and localizing post-treatment biochemical recurrence [10]. Molecular PET, in the post-radical prostatectomy setting, leads to management modifications to prepare patients for salvage radiotherapy by detecting lesions in anatomical locations not typically included in the usual postoperative radiotherapy fields [11]. Finally, PSMA-PET provides more accurate staging for nonmetastatic castrate-resistant PCa, among other applications. In particular, target expression evaluation for PSMA radioligand therapy and target localization for metastasis-directed therapy show potential. Future trials must clarify the potential for this diagnostic tool to translate it into an oncologic benefit [12].

Genetic alterations are associated with differential prognosis and clinical phenotypes in metastatic PCa. Blood biomarkers could assist clinicians in managing patients with localized disease and provide the most robust degree of evidence for predicting more aggressive Pca [13]. Liquid biopsies are valuable as a source of prognostic, predictive, and response biomarkers in PCa. Most clinical applications have been developed in the advanced metastatic setting. These minimally invasive tests can guide diagnosis and treatment selection [14]. However, before therapeutic adoption, newly discovered data on these putative predictive biomarkers must be confirmed in biomarker-driven randomized controlled trials [15]. 

Together, these methods produce risk calculators/nomograms that can predict the risk of developing cancer, the likelihood that the disease will be aggressive, and the likelihood that the patient will respond well to therapy [16,17]. However, we need to learn how to appropriately interpret them and to treat patients while keeping in mind the clinical objectives, such as overall survival, disease recurrence, and quality of life, that the treatment intended to attain. This can only be achieved with sufficiently large studies of patients who are followed up for a long time, even if they are observational studies. This can reduce side effects, expenses, and resource usage while minimizing the danger of over- or under-treating patients.
